# Oil prices, labour market adjustment and dynamic quantile connectedness analysis: evidence from Greece during the crisis

**DOI:** 10.1186/s40008-022-00291-7

**Published:** 2022-12-08

**Authors:** Panagiotis Palaios, Evangelia Papapetrou

**Affiliations:** 1 Department of Accounting, Economics and Finance, The American College of Greece, Athens, Greece; 2 Department of Economics, National and Kapodistrian University of Athens, 1 Sofokleous Str, 10559 Athens, Greece; 3 Economic Research Department, Bank of Greece, Athens, Greece

**Keywords:** Oil prices, Oil price uncertainty, Interest rates, Labour market, Quantile connectedness, C32, E24, E32, Q43

## Abstract

This paper examines the spillover effects transmission mechanism between oil prices, oil price uncertainty and oil price volatility on labour market in Greece, using static and dynamic quantile connectedness methodology (Diebold and Yilmaz Diebold and Yilmaz, Int J Forecast 28:57–66, 2012; Ando et al. Ando T, Greenwood-Nimmo N, Shin Y (2018) ‘Quantile connectedness: Modelling tail behavior in the topology of financial networks’, Working Paper. https://ssrn.com/abstract=3164772.). There is empirical evidence that the oil price variable is the most influential node of the energy variables on hirings and firings, suggesting the endogeneity of the labour market variables. Rolling estimation analysis based on the quantile VAR to capture the volatility spillovers across the whole conditional distribution shows a large variation of the total connectedness index, which is responsive to exogenous adverse and beneficial shocks. Further, our results point to a strong effect due to the COVID-19 pandemic and the state intervention to sustain the pandemic on the labour market. Overall, the analysis reveals a substantial higher time-varying connectedness of the system at the tails of the distribution, indicating that changes in energy markets asymmetrically affect the Greek labour market in recessionary and flourishing states of the economy, rather than normal times.

## Introduction

There is a limited number of studies that have examined the impact of oil prices, oil price uncertainty and oil price volatility on labour market outcomes (Loungani 1986; Papapetrou 2001; Kocaarslan 2019; Koirala and Ma 2020; Kocaarslan et al. 2020). Loungani (1986) shows that there is no aggregate effect of energy price changes on employment. Ferderer (1996) finds that oil price shocks and volatility can help forecast the growth rate of employment. Recently, Kocaarslan (2019) suggests that oil price uncertainty significantly increases unemployment in the U.S. and the response of unemployment to a positive oil price shock is positive, while unemployment decreases in the presence of a negative oil price shock. The study accounts for the presence of asymmetries and shows that the unemployment rate reacts to positive and negative shocks asymmetrically. Koirala and Ma (2020) examine the effects of oil price changes on U.S. aggregate and sectoral employment growth in the presence of time-varying oil price uncertainty. Kocaarslan et al. (2020) study the existence of asymmetric interrelations between oil price uncertainty, oil prices, interest rates and unemployment for the US economy to establish that a rise in oil price causes an increase in unemployment while there is no important effect of oil prices decreases. Furthermore, reduced oil price uncertainty results in a drop in unemployment, while a rise in oil price uncertainty appears not to influence unemployment.

There is a plethora of different methodologies adopted to examine the effects of oil shocks on unemployment. Papapetrou (2001) in a multivariate vector autoregression (VAR) framework examines the dynamic relationship among oil prices, real stock prices, interest rates, real economic activity and employment. Doğrul and Soytas (2010) apply the Toda-Yamamoto estimation procedure to show that real oil prices and interest rates advance the forecasts of unemployment in the long run. Cuestas and Gil-Alana (2018) estimate a nonlinear ARDL (NARDL) model to examine the impact of positive and negative oil price shocks on unemployment. Kocaarslan (2019) uses a GARCH-in-mean VAR model to study the effects of oil price uncertainty on unemployment in the US. Kocaarslan et al. (2020) employ the nonlinear autoregressive distributed lag (NARDL) model to examine the cointegrating relationships and the asymmetric linkages between interest rates, oil prices and oil price uncertainty on unemployment in the US.

Previous research has focused on the direction of spillover effects among employment, oil prices and oil price volatility, relying on average-based estimators that account for average shocks. However, findings based on average shocks that ignore potential heterogeneous effects are unlikely to hold in energy markets and economies. Furthermore, these studies may result in spurious findings due to their inability to capture the time-varying and tail dependence features of the variables. In this paper, we fill this gap in the literature by applying a quantile-based measure of connectedness within the context of Diebold and Yilmaz (2009, 2012, 2014), Diebold et al. (2017) and Ando et al. (2018) that allows to capture the network of connectedness associated with extreme negative and positive disturbances, that is shocks at the lower 1st quantile and the upper 9th quantile of the distribution. In such a case, quantile estimation on a dynamic basis is of vital importance to provide in-depth analysis of the spillover transmission mechanisms along the whole distribution and not only at the mean. In addition, although such an analysis has being applied considering the effects of the pandemic outbreak on financial markets (Bouri et al. 2020) and the returns of the cryptocurrency markets (Bouri et al. 2021) to the best of our knowledge, it is missing in the oil shock and labour market interrelationship analysis.

This paper’s contribution to the existing literature is as follows. First, using daily observations for Greece for the period March 1, 2013 to October 31, 2020 the analysis extends our understanding on the potential evidence of the spillover between labour market and oil prices. Even though previous research has documented the effect of oil price changes and oil price uncertainty on unemployment, there is no study examining the spillover of oil prices on labour market outcomes. To account for this shortcoming, we estimate the interrelations between oil prices, oil price uncertainty, interest rates and hirings and firings in the labour market. Second, the analysis explores the spillover between oil prices, oil price uncertainty, interest rates and labour market outcomes in Greece. Greece since 2010 has implemented a bold economic reform and entered adjustment programmes that have eliminated macroeconomic imbalances. The cost of the adjustment programme, in terms of output and unemployment losses, has been immense, as GDP dropped by almost 25% and unemployment rate soared to 27.5%. Further significant labour market reforms were introduced that included, among others, measures to decentralize the wage bargaining system, reduce employment protection by lowering firing costs and increase working time flexibility (Kosma et al. 2017; Papapetrou and Tsalaporta 2021). Moreover, Greece is a medium-sized economy heavily dependent on oil and more vulnerable than other economies to changes in the international oil market. The economy relies heavily on energy imports and has only minor domestic reserves of oil. Greece energy dependency was 68.9% in 2019, well above the European average (EU-27: 60.6 percent). Oil is mostly used in transport (64%), residential sector (14%) and industry (12%) (2017). Third, the study investigates the connectedness and spillover effects between labour market, oil prices, oil price uncertainty and interest rates employing the static and dynamic connectedness framework proposed by Diebold and Yilmaz (2009, 2012, 2014). Contrary to other correlation and causal-based approaches, connectedness analysis allows developing a unified methodology for measuring volatility spillover effects at a variety of levels, from pairwise through system-wide, based on forecast variance decompositions from vector autoregressions (VARs). Although recent empirical analysis has explored the effect of oil price shocks on stock and sovereign bond markets using the approach of Diebold and Yilmaz (Benlagha and Hemrit 2022), to the best of our knowledge, the connectedness and spillover effects between oil prices and the labour market in Greece are missing. Greece serves as an example and the conclusions drawn on the dynamic interrelations between these variables could be indicative of conditions in other medium-sized open economies. Fourth, moving beyond conditional mean estimation analysis, this paper utilizes a quantile-based approach of Diebold and Yilmaz (2012) and further developed by Ando et al. (2018) to derive the spillover effects variation and the net total directional connectedness of the variables across the whole distribution. Such a differentiation in the quantile connectedness and especially between lower and upper quantiles permits to identify between extreme negative shocks (left-tail dependence) and extreme positive shocks (right-tail dependence) and to provide an in-depth analysis of the transmission mechanisms along the distribution. Lastly, our empirical results might be useful to other countries sharing comparable features.

The rest of this paper is organized as follows. Sect. 2 presents a review of previous studies on the empirical evidence of oil price changes, oil price volatility and the labour market. Sect. 3 deals with methodological issues and the data employed in the empirical analysis. Sect. 4 presents the empirical findings. Finally, in Sect.  5 the conclusions of the analysis are summarized.

## The labour market and oil prices: evidence from the literature

The impact of oil price shocks on macroeconomic variables is of vital importance for the overall performance of the economy as it affects both the demand and supply side of the market. Considering the theoretical backgrounds among oil price and macroeconomic variables, Hamilton (2008) states that it is logical to expect that oil price changes imply macroeconomic effects on the inflation rate, as oil price shocks are a crucial determinant of the monetary policy. On this important issue Hooker (2002), in a seminal contribution which is conducted in an asymmetric framework, provides empirical support in favour of the view that changes in oil prices were an important component of US inflation rate only before 1981. Kilian (2008) states that in order to achieve an in-depth analysis of the effects of energy price shocks on the economic activity it is essential to distinguish between the effects of demand shocks and supply shocks in the energy markets. Gadea et al. (2016) examine the impact of oil price shocks on GDP growth and find consistent evidence that the effect of a negative oil price shock on GDP growth is more intense when the increase in oil prices are larger. Mohaddes et al. (2017) confirm the existence of nonlinearities in the macroeconomic effects of oil price changes as they find empirical evidence that after an oil shock the equilibrium is restored with a low pace accompanied by intense volatility in the level of prices. Recently, Chan and Dong (2022) examine the impact of oil price volatility on the unemployment rates using a DSGE model. They find that an unexpected increase in the volatility of oil prices implies a persistent effect on the unemployment rates.

Various studies have examined the role of oil prices and oil price uncertainty for variations in the labour market for developed countries. Loungani (1986) shows that there is no aggregate effect of energy price changes on employment and oil price increases in the 1950s and 1970s increase unemployment and account for an unusual amount of labour reallocation across industries. Gisser and Goodwin (1986) display that oil price shocks affect a set of macro variables including employment and detect a relationship between the crude oil price and employment. Keane and Prasad (1996) use micro panel data to study the effects of oil price changes on employment and real wages, at the aggregate and industry level in the US. Gil-Alana and Brian Henry (2003) using fractional integration methods for the UK show that oil prices, interest rates and unemployment are integrated.

Recently, Herrera and Karaki (2015) examine the effect of oil price innovations on U.S. job flows in manufacturing accounting for symmetric and asymmetric responses. They show that positive innovations lead to a decline in net employment and an increase in job reallocation. Herrera et al. (2017) estimate a factor augmented vector autoregressive model to examine the effect of oil price shocks on private job flows as well as on industry-level job creation and destruction. After an unanticipated oil price decrease in the first year, they show that there is a reduction in job creation and an increase in job destruction in oil and gas extraction and support activities for mining. However, other industries (construction, manufacturing and services) show an increase in the net employment change. Karaki (2018) examines the effect of oil price innovations on manufacturing job flows across U.S. states estimating a nonlinear structural equation model. The author documents asymmetries in the responses of job flows to positive and negative oil price innovations and that oil price shocks have limited regional allocative effects.

Kocaarslan (2019) examines the effects of oil price uncertainty and oil price shocks on U.S. unemployment rate using a GARCH-in-mean VAR model for the period 1974:q2–2017:q4. The findings suggest that oil price uncertainty significantly increases the unemployment rate in the U.S. economy and oil price uncertainty increases the rise in the unemployment rate. Furthermore, the study shows that the unemployment rate reacts to positive and negative shocks asymmetrically. Besides, Koirala and Ma (2020) examine the effects of oil price changes on U.S. aggregate and sectoral employment growth in the presence of time-varying oil price uncertainty. The authors estimate a bivariate GARCH-in-Mean VAR model employing data over the period 1974:02–2018:11. They document that an increase in oil prices reduces total employment growth mainly in the private sector; however, employment in the public sector is unaffected. Finally, the authors show that employment growth responds asymmetrically to positive and negative oil price shocks. Kocaarslan et al. (2020) examine the existence of asymmetric interrelations between oil prices, oil price uncertainty, interest rates and unemployment in the US market. Utilizing the nonlinear autoregressive distributed lag (NARDL) approach the authors convey that a rise in oil price causes an increase in unemployment, whereas there is no significant effect of a decrease in oil prices. However, reduced oil price uncertainty results in a drop in unemployment, while a rise in oil price uncertainty does not have an effect.

However, fewer studies have examined the impact of oil prices on labour market outcomes for other countries except the U.S. Doğrul and Soytas (2010) examine the causality between unemployment, oil and real interest rate in Turkey over the period 2005:01–2009:08. This paper shows that in the long run, the real price of oil and interest rate improve the forecasts of unemployment. Cuestas and Gil-Alana (2018) examine the positive and negative movements of oil prices on unemployment rates in Central and Eastern Europe over the period 2000:1–2015:4. They demonstrate that although oil prices and unemployment are not correlated to a great extent in the short run, oil price shocks move in the same direction with the natural rate of unemployment.

Empirical findings for Greece are limited and focuses on the interdependence of oil price changes, stock returns and economic activity or employment (Papapetrou 2001, 2013). Papapetrou (2001) using a VAR approach among oil prices, real stock prices, interest rates, real economic activity and employment in Greece shows that oil price changes affect negatively real economic activity and employment and oil price shocks explain a significant proportion of the fluctuations in output growth and employment growth.

Recently, Betcherman et al. (2020) examine the short-term labour market impacts of the COVID-19 lockdown in Greece. They show, without accounting for possible explanations of these patterns, that the measures taken by the government to alleviate the effects of the pandemic prohibited layoffs and as a result flows into unemployment were inhibited. Raifu et al. (2020) examine the relationship between oil price changes and unemployment in Nigeria applying linear ARDL and NARDL methodologies. They conclude that changes in oil prices have a statistically insignificant effect on the unemployment in the short term. However, in the long term, they find strong evidence in favour of asymmetric effects and document that an increase in oil prices leads to an increase in unemployment, while a decrease in oil prices has a statistically insignificant reducing effect. Michieka et al. (2022) examine the relationship between changes in employment in the coal industry and unemployment. Their sample contains 20 industries in 10 US states that produce coal, covering the period 2001–2018. They find that, in the long run, migration of coal workers, due to energy transition policies, leads to a reduction in the wages of many sectors of the economy. Chen et al. (2022) examine the impact of policy intervention, through fuel pricing, in household energy transition accounting for the labour market, focusing on Sichuan Province of China. They find evidence that due to imperfect labour market the effectiveness of price incentives has a low effect on household energy transition, stressing the need for the development of a social safety net to tackle market failures. Cheratian et al. (2021) research the impact of oil prices on unemployment in an asymmetric NARDL framework, focusing on the Middle East and North Africa region, over the period 1991–2017. They show that in the long run, positive changes of oil prices lead to an increase in the unemployment in the region while the corresponding effect of a decrease in oil prices on the unemployment it is, as expected, negative, but of lower intense. Recently, Michieka and Gearhart (2022) examine the nexus between oil prices and wages in 15 top oil-producing counties in the USA over the period 2001 to 2018. Results indicate that in the long run, a positive oil price shocks granger causes wages increases in all sectors, with the largest increase in the manufacturing sector. County level analysis results from the NARDL modelling approach suggest that there is no consistent effect on wages following oil price shocks in the long and short run in the total economy.

## Econometric framework and data

To examine the pairwise relationship between the variables in consideration, we frame our empirical strategy in two stages. Initially, we examine the spillover effects of our system by applying the static and dynamic connectedness analysis developed by Diebold and Yilmaz (2009, 2012, 2014). Second, following Ando et al. (2018) we apply the quantile connectedness methodology, which allows to have a more detailed analysis of the spillover effects across the whole distribution of the variables.

### Static and dynamic connectedness analysis

To estimate the degree of connectedness between the variables of our model we initially apply the methodology developed by Diebold and Yilmaz (2009, 2012, 2014) as follows. We consider a covariance stationary N-process Var(p), $y_{t} = \sum\nolimits_{i = 1}^{p} {\Phi_{i} y_{t - i} + \varepsilon_{t} }$, where ${\varepsilon }_{t}$~iid (0,${\sigma }_{\varepsilon }^{2}$). The moving average representation is given by ${y}_{t}={\rm A}(L){u}_{t}$, where ${\rm A}\left(L\right)$ is an n x n infinite lag polynomial matrix of coefficients and ${\rm E}\left({u}_{t}{u}_{t}^{^{\prime}}\right)=I$. The corresponding *h*-step ahead generalized forecast error variance decompositions by ${\theta }_{ij}^{g}(h)$, for $h=\mathrm{1,2},\dots n,$ is as follows:

1 $$\theta_{ij}^{g} \left( h \right) = \frac{{\sigma_{jj}^{ - 1} \mathop \sum \nolimits_{h = 0}^{H - 1} (e_{i}^{^{\prime}} A_{h} \sum e_{j} )^{2} }}{{\mathop \sum \nolimits_{h = 0}^{H - 1} \left( {e_{i}^{^{\prime}} A_{h} \sum A_{h}^{^{\prime}} e_{i} } \right)}},$$

where ${\sigma }_{jj}$ is the standard deviation of the error term for the j-th equation, ${e}_{j}$ is a selection vector with j-th element equal to one and zero otherwise, $\Sigma$ is the variance matrix for the error vector $\varepsilon$, and ${A}_{h}$ is the coefficient matrix multiplying the $h$-lagged error vector.

The total volatility spillover index (S) is estimated as the following *h*-step ahead forecast relative to total forecast error variation:

2 $$S^{g} \left( h \right) = \frac{{\mathop \sum \nolimits_{{i,j = 1,{ }i \ne j}}^{n} \tilde{\theta }_{ij}^{g} \left( h \right)}}{{\mathop \sum \nolimits_{i,j = 1}^{n} \tilde{\theta }_{ij}^{g} \left( h \right)}}100 = 1/n\mathop \sum \limits_{{i,j = 1,{ }i \ne j}}^{n} \tilde{\theta }_{ij}^{g} \left( h \right)\cdot100,$$

where ${\widetilde{\theta }}_{ij}^{g}\left(h\right)=\frac{{\theta }_{ij}^{g}(h)}{\sum_{j=1}^{N}{\theta }_{ij}^{g}(h)}$, are the ${\theta }_{ij}^{g}(h)$ normalized h-step ahead error variance decompositions.

The directional volatility spillovers to variable *i* from all other variables are given by

3 $$S_{i \leftarrow others}^{g} \left( h \right) = \frac{{\mathop \sum \nolimits_{{i,j = 1,{ }i \ne j}}^{n} \tilde{\theta }_{ij}^{g} \left( h \right)}}{{\mathop \sum \nolimits_{i,j = 1}^{n} \tilde{\theta }_{ij}^{g} \left( h \right)}}100 = 1/n\mathop \sum \limits_{{i,j = 1,{ }i \ne j}}^{n} \tilde{\theta }_{ij}^{g} \left( h \right)\cdot100.$$

The directional volatility spillovers by variable *i* to all other variables are given by

4 $$S_{others \leftarrow i}^{g} \left( h \right) = \frac{{\mathop \sum \nolimits_{{i,j = 1,{ }i \ne j}}^{n} \tilde{\theta }_{ij}^{g} \left( h \right)}}{{\mathop \sum \nolimits_{i,j = 1}^{n} \tilde{\theta }_{ij}^{g} \left( h \right)}}100 = 1/n\sum\nolimits_{i,j = 1,i \ne j}^{n} {\tilde{\theta }_{ij}^{g} \left( h \right)\cdot100} .$$

Consequently, the net volatility spillover effect from market *i* to all other markets *j* is given from

5 $$S_{i}^{g} \left( h \right) = S_{others \leftarrow i}^{g} \left( h \right) - S_{i \leftarrow others}^{g} \left( h \right).$$

The net pairwise spillover effect between variables *i* and *j* is the difference between the gross validity shocks transmitted from variable *i* to variable *j* and those transmitted form *variable j to i and* is given by

6 $$S_{ij}^{g} \left( h \right) = \left[ {\frac{{\tilde{\theta }_{ji}^{g} \left( h \right)}}{{\mathop \sum \nolimits_{i,k = 1}^{N} \tilde{\theta }_{ik}^{g} \left( h \right)}} - \frac{{\tilde{\theta }_{ij}^{g} \left( h \right)}}{{\mathop \sum \nolimits_{j,k = 1}^{N} \tilde{\theta }_{jk}^{g} \left( h \right)}}} \right]100 = \frac{{\tilde{\theta }_{ij}^{g} \left( h \right) - \tilde{\theta }_{ji}^{g} \left( h \right)}}{n}100.$$

After examining the connectedness over the entire sample period, we derive the associated *h*-step ahead forecast error variance decomposition, by employing a rolling window, to assess the spillover effects variation over time.

### Quantile connectedness analysis

Further, we follow Ando et al. (2018) to develop a quantile connectedness framework for the variables of our system. Quantile connectedness approach allows examining the spillover effects between the variables of the system at a given conditional quantile, *τ* ∈ (0,1) (Antonakakis et al. 2019; Chatziantoniou and Gabauer 2021; and Bouri et al. 2021).

The corresponding measures of the network topology at the τ-th quantile are given by

7 $$O_{i \leftarrow i,\left( \tau \right)}^{\left( h \right)} = \theta_{i \leftarrow i,\left( \tau \right)}^{\left( h \right)},$$

where ${O}_{i\leftarrow i,(\tau )}^{(h)}$ is the proportion of the *h*-step ahead forecast error variance of the *i*-th variable, at the τ-th quantile, that can be attributed to shocks to itself, called own variance share,

8 $$F_{i \leftarrow \cdot,\left( \tau \right)}^{\left( h \right)} = \mathop \sum \limits_{j = 1,j \ne i}^{m} \theta_{i \leftarrow j,\left( \tau \right)}^{\left( h \right)},$$

where ${F}_{i\leftarrow \cdot ,(\tau )}^{(h)}$ measures the total spillover from the system to variable *i,* at the τ-th quantile,

9 $$T_{\cdot \leftarrow i,\left( \tau \right)}^{\left( h \right)} = \mathop \sum \limits_{j = 1,j \ne i}^{m} \theta_{j \leftarrow i,\left( \tau \right)}^{\left( h \right)},$$

where ${T}_{\cdot \leftarrow i,(\tau )}^{(h)}$ measures the total spillover from variable *i,* to the system, at the τ-th conditional quantile,

10 $${\rm N}_{i \leftarrow i,\left( \tau \right)}^{\left( h \right)} = T_{\cdot \leftarrow i,\left( \tau \right)}^{\left( h \right)} - F_{i \leftarrow \cdot,\left( \tau \right)}^{\left( h \right)},$$

where ${\rm N}_{i\leftarrow i,(\tau )}^{(h)}$ measures the net directional connectedness of variable *i*, at the τ-th conditional quantile,

11 $$TSI_{\tau }^{h} = m^{ - 1} \sum\nolimits_{i = 1}^{m} {F_{i \leftarrow \cdot,\left( \tau \right)}^{h} },$$

where $TSI_{\tau }^{h}$ is the total spillover index (TSI) at the τ-th conditional quantile.

### Data

The data employed in this paper are daily frequency from March 1, 2013 to October 31, 2020. Availability of the labour market variables determines the empirical analysis since the labour market outcomes are not available prior to March 2013. In total, we have 2801 daily observations for each series. For crude oil price data, Brent crude oil spot prices (oil) in euro terms are collected from the Energy Information Administration (EIA). The two labour market variables, hirings *(hr)* and firings *(fr*), are collected from the ERGANI database published by the Ministry of Labour and Social Affairs. The crude oil price volatility index *(ovx)* obtained from options markets as a proxy for oil price uncertainty is employed. The *ovx* measures 30-day volatility expectations in the United States Oil Fund option prices. We use the yield on 10-year Greek bonds *(bnd)* to evaluate the impact of monetary policy on the labour market. The interest rate series is sourced from the Bank of Greece. Finally, following Hamilton (2003) and Kocaarslan et al. (2020) we acquire the conditional volatility of oil price changes, *ovxcv*, as a proxy for uncertainty, using a GARCH (1,1) specification. The conditional variance equation of the GARCH (1, 1) model of Bollerslev (1986) is specified as follows:

12 $$\sigma_{t}^{2} = \beta_{0} + \beta_{1} \varepsilon_{t - 1}^{2} + \beta_{2} \sigma_{t - 1}^{2},$$

where the conditional variance in Eq. (12) is a function of the terms: (i) the mean, ${\beta }_{0}$; (ii) news about volatility from the previous period, measured as the lag of the squared residuals from the mean equation, ${\varepsilon }_{t-1}^{2}$ (ARCH term); (iii) the last period’s forecast error variance, ${\sigma }_{t-1}^{2}$ (GARCH term). The estimates of the conditional variance equation are reported in Table 1.

**Table 1:** Estimated coefficients of the GARCH (1,1) model

Variable	${\upbeta }_{0}$	${\upbeta }_{1}$	${\upbeta }_{2}$
*oil*	11.761***(0.253)	0.922***(0.030)	0.151***(0.014)

^*^, **, *** denote significance at 10%, 5% and 1% level, respectively. Standard erros are in parentheses

**Fig. 1:**
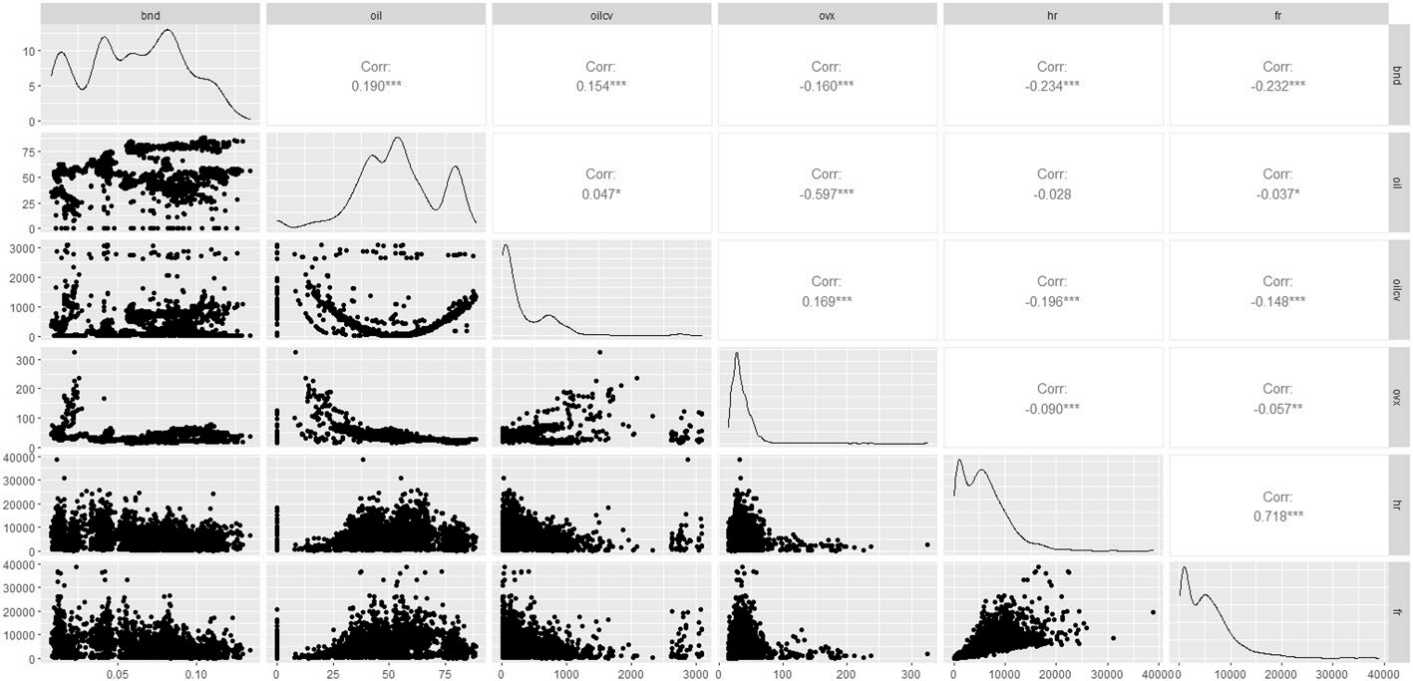
Scatterplot matrices, Pearson correlation coefficients and variables distribution

Table 2 provides the summary statistics of the variables and a conventional unit root test. Statistics from the augmented Dickey-Fuller (ADF) test specify that all series are stationary. The statistical properties of the series provide evidence of nonnormal distribution, since the kurtosis statistic for *hr, fr, ovx, oilcv* and *oil* variables is higher than 3 (leptokurtic distribution), while for *bnd* series the kurtosis statistics is lower than 3 (platykurtic distribution). Figure 1 displays scatterplot matrices (left part), Pearson correlation coefficients (right part) and variables distribution (diagonal). Overall, the graphical representation of the variables distribution confirms the previous indications of a nonnormal distribution, while Pearson correlation coefficients show the existence of statistically significant correlation between all the pairwise relationships of our system.

**Table 2:** Summary statistics for the variables and JB, ADF test

Variable	obs	mean	median	standard deviation	skewness	kurtosis	Jarque–bera	ADF
*hr*	2802	5793	5232	4362.09	1.19	5.68	1508.4***	− 4.73***
*fr*	2802	5430	4684	4853.52	1.83	9.06	5872***	− 3.77***
*ovx*	2802	36.63	31.79	21.84	4.50	34.46	125028***	− 3.05***
*oilcv*	2802	361.60	153.63	491.80	2.75	13.05	15322***	− 4.87***
*oil*	2802	53.74	53.21	17.88	− 0.30	3.25	49.55***	− 2.70**
*bnd*	2802	0.061	0.062	0.03	− 0.031	2.03	110.9***	− 1.90**

Table 2 provides the results of the Augmented Dickey-Fuller (ADF) test and it is conducted with an inteercept and lag length selected by Akaike Information Criterion (AIC)

^*^, **, *** denote significance at 10%, 5% and 1% level, respectively

The normality properties of the series are examined applying the Jarque and Bera (1980) conventional normality test and the quantile-mean covariance (QC) test developed by Bera et al. (2016). The conventional test results, presented in Table 2, reject the null hypothesis that our series are normally distributed. Further, QC normality test results, presented in Table 3, indicate an asymmetric behaviour of all series distribution, as the null hypothesis of normality is rejected in all cases.

**Table 3:** Quantile-mean covariance (QC) test of normality

		ε = 0.001	ε = 0.01	ε = 0.05	ε = 0.10	ε = 0.15	ε = 0.20
*hr*	${\rm T}_{1n}$	9.008***	9.008***	9.008***	9.008***	1.834***	0.751***
	${\rm T}_{2n}$	81.160***	81.160***	81.160***	81.160***	3.365***	0.564***
	${\rm T}_{3n}$	5.966***	5.884***	4.931***	1.887***	0.266***	0.180***
*fr*	${\rm T}_{1n}$	11.375***	11.375***	11.375***	11.375***	9.968***	2.088***
	${\rm T}_{2n}$	129.405***	129.405***	129.405***	129.405***	99.371***	4.363***
	${\rm T}_{3n}$	12.976***	12.829***	11.672***	8.546***	2.901***	1.322***
*ovx*	${\rm T}_{1n}$	12.981***	12.981***	12.981***	12.981***	12.981***	12.386***
	${\rm T}_{2n}$	168.508***	168.508***	168.508***	168.508***	168.508***	153.429***
	${\rm T}_{3n}$	47.495***	47.237***	46.272***	43.403***	37.200***	28.295***
*oilcv*	${\rm T}_{1n}$	16.418***	16.418***	16.418***	16.418***	16.418***	16.418***
	${\rm T}_{2n}$	269.559***	269.559***	269.559***	269.559***	269.559***	269.559***
	${\rm T}_{3n}$	34.647***	34.415***	33.317***	30.398***	24.442***	15.123***
*oil*	${\rm T}_{1n}$	4.627***	4.627***	4.627***	4.627***	3.194***	1.719***
	${\rm T}_{2n}$	21.409***	21.409***	21.409***	21.409***	10.206***	2.957***
	${\rm T}_{3n}$	2.636***	2.586***	2.101***	1.712***	0.685***	0.284***
*bnd*	${\rm T}_{1n}$	4.653***	4.653***	3.933***	3.933***	2.837***	2.837***
	${\rm T}_{2n}$	21.658***	21.658***	15.473***	15.473***	8.052***	8.052***
	${\rm T}_{3n}$	3.306***	3.291***	2.645***	2.233***	1.698***	1.605***

^*^, **, *** denote significance at 10%, 5% and 1% level, respectively. T1, T2, T3 refer to Bera et al. (2016) statistics: $$T_{1n} \,: = \,\begin{array}{*{20}c} {\sup } \\ {\tau ET} \\ \end{array} \left| {\mathop {C_{n} }\limits^{ \wedge } \left( \tau \right)} \right|,\,T_{2n} : = \,\begin{array}{*{20}c} {\sup } \\ {\tau ET} \\ \end{array} \mathop {C_{n} }\limits^{ \wedge } \left( \tau \right)^{2} ,\,T_{3n} : = \,\int\limits_{\tau ET} {\mathop {C_{n} }\limits^{ \wedge } \left( \tau \right)^{2} } dr,$$ where $\mathop {C_{n} }\limits^{ \wedge } \left( \tau \right)$ is the quantile-mean covariance (QC) function, which is the asymptotic covariance between the sample quantiles and the sample mean

## Empirical results

The empirical analysis is conducted as follows. First, the average (conditional mean) and conditional median (τ = 0.5) values of the total spillover index (TSI) are presented (Sect. 4.1). Second, the volatility spillovers across the whole conditional distribution, with emphasis on the connectedness at the lower and upper tails are introduced (Sect. 4.2). As a next step, we perform a rolling analysis based on the quantile VAR to examine the volatility spillovers across the whole conditional distribution (Sect. 4.3.1) and the evolution of time-varying net total directional connectedness (Sect. 4.3.2).

### Conditional mean and median spillovers

Table 4 reports the connectedness measure estimated at the conditional mean. The total connectedness of the model is 26.6%, showing that there is significant shock transmission to the system and the variables are not independent from each other. The oil price variable (*oil*) is the most influential node of the energy variables on hirings (hr), as it explains 12.3% of hirings variation, followed by the conditional volatility of oil price changes (*oilcv*) (4.2%) and the crude oil volatility index (*ovx*) (3.9%), as well as on firings as it explains 11.0% of the firings variation, followed by crude oil volatility index (*ovx*) (4.1%) and the conditional volatility of oil price changes (*oilcv*) (3.8%). As expected, there is evidence in favour of a high degree of interdependence between hirings and firings which explains the contribution of these two variables to the system. Specifically, hirings explains 26.6% of firings variations while the corresponding contribution of firings to hirings’ variation is 32.9%. The corresponding pairwise transmission mechanism is from firings to hirings (${\widehat{S}}_{hr, fr}={\widehat{S}}_{fr \leftarrow hr}-{\widehat{S}}_{hr \leftarrow fr}$= 26.6–32.9 = − 6.3), namely in the relationship between the two variables, firings is the driving force. The corresponding pairwise transmission mechanisms between hirings and the energy variables of the system are from crude oil volatility index to hirings (${\widehat{S}}_{hr, ovx}={\widehat{S}}_{ovx \leftarrow hr}-{\widehat{S}}_{hr \leftarrow ovx}$= 0.0–3.9 = − 3.9), from conditional volatility of oil price changes to hirings (${\widehat{S}}_{hr, oilcv}={\widehat{S}}_{oilcv \leftarrow hr}-{\widehat{S}}_{hr\leftarrow oilvc}$= 0.2–4.2 = − 4.0),) and from oil price variable to hirings (${\widehat{S}}_{hr, oil}={\widehat{S}}_{oil \leftarrow hr}-{\widehat{S}}_{hr \leftarrow oil}$= 1.9–12.3 = − 10.4), namely the energy variables act as the driving force in their relationship with hirings, with the latter being the endogenous variable. In total, the energy variables can explain 20.4% of hirings variance, from which 12.3% is the contribution of oil price variable. This suggests that the oil price variable is the most influential node of the energy variables on hirings. Similar conclusions can be drawn from the examination of the pairwise transmission mechanism between firings and the energy variables. The corresponding pairwise transmission mechanism is from the crude oil volatility index to firings (${\widehat{S}}_{fr, ovx}={\widehat{S}}_{ovx \leftarrow fr}-{\widehat{S}}_{fr \leftarrow ovx}$= 0.1–4.1 = − 4.0), from the conditional volatility of oil price changes to firings (${\widehat{S}}_{fr, oilcv}={\widehat{S}}_{oilcv \leftarrow fr}-{\widehat{S}}_{fr \leftarrow oilvc}$= 0.7–3.8 = − 3.1) and from the oil price variable to firings (${\widehat{S}}_{fr, oil}={\widehat{S}}_{oil \leftarrow fr}-{\widehat{S}}_{fr \leftarrow oil}$= 3.6–11.0 = − 7.4). In total, the energy variables can explain 18.9% of firings variance, from which 11.0% is the contribution of the oil price variable. This in turn means that the oil price variable is the most influential node of the energy variables on firings, as well. In net terms, total connectedness takes place from the energy variables (*ovx, oilcv, oil*) to hirings (${\widehat{S}}_{c}={\widehat{S}}_{energy \leftarrow hirings}-{\widehat{S}}_{hirings \leftarrow energy}$= 2.1%–20.4% = − 18.3%) and firings (${\widehat{S}}_{firings}={\widehat{S}}_{energy \leftarrow firings}-{\widehat{S}}_{firings \leftarrow energy}$= 4.4%–18.9% = − 14.5%), which implies that the energy variables are net transmitters, acting as exogenous variables in their relation with hirings and firings, while these two variables are endogenous.^1^ Our findings are in line with Doğrul and Soytas (2010), Koirala and Ma (2020) and Kocaarslan et al. (2020), showing that the conditional volatility of crude oil prices and oil prices affect unemployment. For Greece, the results are in line with the findings of Papapetrou (2001) reporting that oil price changes affect negatively real economic activity and employment. On explanation for the consistency of the findings is that Greece relies on oil and has not developed adequate alternative energy-saving technologies and as a result has a very high tolerance of oil price changes and oil price volatility (Papapetrou 2013).

**Table 4:** System connectedness evaluated at the conditional mean

	hr	fr	ovx	oilcv	oil	bnd	FROM
hr	46.4	32.9	3.9	4.2	12.3	0.2	53.6
fr	26.6	54.2	4.1	3.8	11.0	0.2	45.8
ovx	0.0	0.1	98.1	0.5	0.9	0.3	1.9
oilcv	0.2	0.7	5.2	80.0	13.0	0.9	20.0
oil	1.9	3.6	1.6	28.4	62.4	2.1	37.6
bnd	0.1	0.1	0.6	0.0	0.0	99.1	0.9
Contribution TO others	28.8	37.5	15.4	37.0	37.3	3.7	**26.6**
NET directional connectedness	− 24.8	− 8.3	13.5	17.0	− 0.3	2.9	

The *ij*-entry of the upper left 6 × 6 variable submatrix gives the *ij*-th pairwise directional connectedness. The rightmost (FROM) column gives total directional connectedness from all other variables to *i* variable. The bottom (TO) row gives total directional connectedness to all other variables from *j* variable. The bottommost (NET) row gives the differencing total directional connectedness (to–from). The bottom right element, in bold, is total connectedness

Table 5 reports the total connectedness for all the variables of the system, evaluated at the median of the conditional distribution (τ = 0.5). The TSI has increased significantly relative to the corresponding connectedness index evaluated at the mean, from 26.6% to 38.7%, revealing an even higher interconnection between the variables. Contrary to mean analysis, the conditional volatility of oil price changes is the most influential node of the energy variables on hirings, as it explains 7.7% of hirings variation, followed by oil price variable (7.3%) and the crude oil volatility index (2.3%), as well as on firings as it explains 9.2% of the firings variation, followed by oil price variable (8.2%) and the crude oil volatility index (1.7%). As in the mean analysis, there is a high degree of interdependence between hirings and firings which explains the contribution of these two variables to the system. Specifically, hirings explains 24.6% of firings variations, while the corresponding contribution of firings to hirings’ variation is 28.6%. The corresponding pairwise transmission mechanism is from firings to hirings (${\widehat{S}}_{hr, fr}={\widehat{S}}_{fr \leftarrow hr}-{\widehat{S}}_{hr \leftarrow fr}$= 24.6%–28.6% = − 4.0%), namely in the relationship between the two variables, firings is confirmed once more to be the driving force. The corresponding pairwise spillover transmission mechanisms between hirings and firings and the energy variables of the system are, as in the mean analysis, from the energy variables to hirings and firings, which in turn implies that the energy variables act as the driving force. The total contribution of the energy variables to hirings (17.3%) and firings (19.1%) remains relative steady, from which 7.7–9.2%, respectively, is the contribution of the conditional volatility of oil price changes (oilcv), which in turn means that it is the most influential node of the energy variables. In net terms, total connectedness takes place from the energy variables (*ovx, oilcv, oil*) to hirings (${\widehat{S}}_{c}={\widehat{S}}_{energy \leftarrow hirings}-{\widehat{S}}_{hirings \leftarrow energy}$= 3.3%–17.3% = − 14.0%) and firings (${\widehat{S}}_{firings}={\widehat{S}}_{energy \leftarrow firings}-{\widehat{S}}_{firings \leftarrow energy}$= 3.7%–19.1% = − 15.4%), which implies that the energy variables are net transmitters, acting as exogenous variables in their relation with hirings and firings, while these two variables are endogenous. Overall, it is shown that both the mean and median spillover indices, which are measures of the average connectedness of the system, display broadly similar behaviour, a finding in line with Ando et al. (2018) showing that the spillover indices at the conditional mean and median follow similar patterns.

**Table 5:** System connectedness evaluated at the median (τ = 0.5)

	hr	fr	ovx	oilcv	oil	bnd	FROM
hr	51.9	28.6	2.3	7.7	7.3	2.2	48.1
fr	24.6	54.8	1.7	9.2	8.2	1.4	45.2
ovx	1.6	1.7	68.5	9.8	16.1	2.2	31.5
oilcv	0.9	1.0	5.9	51.1	40.6	0.5	48.9
oil	0.8	1.0	5.1	17.4	75.1	0.7	24.9
bnd	0.8	1.3	10.5	11.1	9.9	66.4	33.6
Contribution TO others	28.7	33.6	25.5	55.2	82.2	7.0	**38.7**
NET directional connectedness	− 19.4	− 11.5	-6.0	6.3	57.2	− 26.7	

The *ij*-entry of the upper left 6 × 6 variable submatrix gives the *ij*-th pairwise directional connectedness. The rightmost (FROM) column gives total directional connectedness from all other variables to *i* variable. The bottom (TO) row gives total directional connectedness to all other variables from *j* variable. The bottommost (NET) row gives the differencing total directional connectedness (to–from). The bottom right element, in bold, is total connectedness

### Connectedness measures across the distribution

**Fig. 2:**
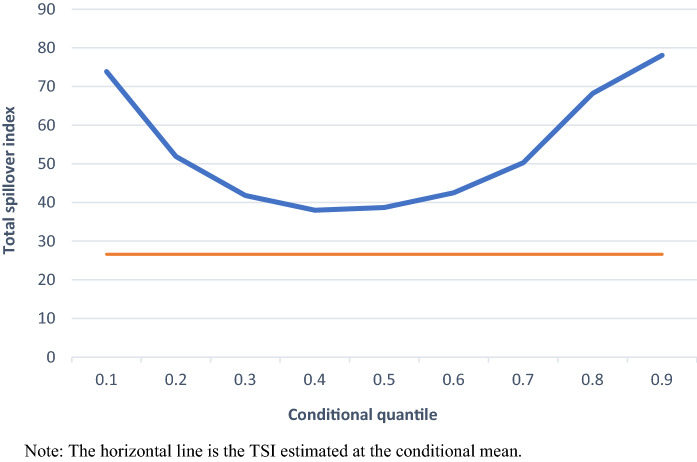
Total Spillover Index (TSI) along the conditional distribution. The horizontal line is the TSI estimated at the conditional mean

Next, we apply quantile connectedness analysis which allows spillover effects of the covariates to differ across various quantiles of the distribution and provide the connectedness measures at different points of the conditional distribution. Figure 2 reports the variation of the total spillover index (TSI) evaluated at various quantiles and captures the impact of negative and positive shocks of different magnitude. We interpret an increase (decrease) of the TSI as evidence of an increase (decrease) in the sensitivity of the variables due to the corresponding positive and/or negative shock, namely an increase (decrease) of the spillover effects transmission among our variables. The TSI exhibits a large variation across the quantiles, providing evidence in favour of excess spillover effects during negative (left-tail-dependence) and positive (right-tail-dependence) shocks. These results are in line with Bouri et al. (2021) reporting that TSI is higher after both positive and negative shocks but contradict the findings of Antonakakis et al. (2019) and Ando et al. (2018) that provide evidence that total connectedness rises after adverse shocks. The analysis shows that the TSI ranges between 38% (τ = 0.4) and 78.1% (τ = 0.9). Extreme negative shocks (τ = 0.1) lead to an increase of the index up to 73.9%, unveiling a gradual decrease as negative shocks decrease in magnitude. TSI reaches its lowest values at the fourth quantile (38.0%) and fifth quantile (38.7%), displaying a gradual increase as the intensity of positive shocks increases. Consequently, as the energy variables are net transmitters of spillover effects in their relationship with firings and hirings, namely they act as exogenous variables, we show that the impact of the energy variables on labour market is of greater magnitude after positive and negative shocks.

### Time-varying analysis

In this section we turn our analysis to dynamic quantile connectedness to measure the volatility spillovers across the conditional distribution, over time. We use a fixed window length of 270 days and a 40-step ahead forecast horizon.^2^ We frame our analysis in two steps. First, we examine the time-varying evolution of the TSI, which is a modification of the various spillover effects in a single index. Second, we examine the time-varying evolution of the net total directional connectedness, which is a measure of the net spillover effect of each variable on the system.

#### Total Spillover Index: dynamic quantile evolution

**Fig. 3:**
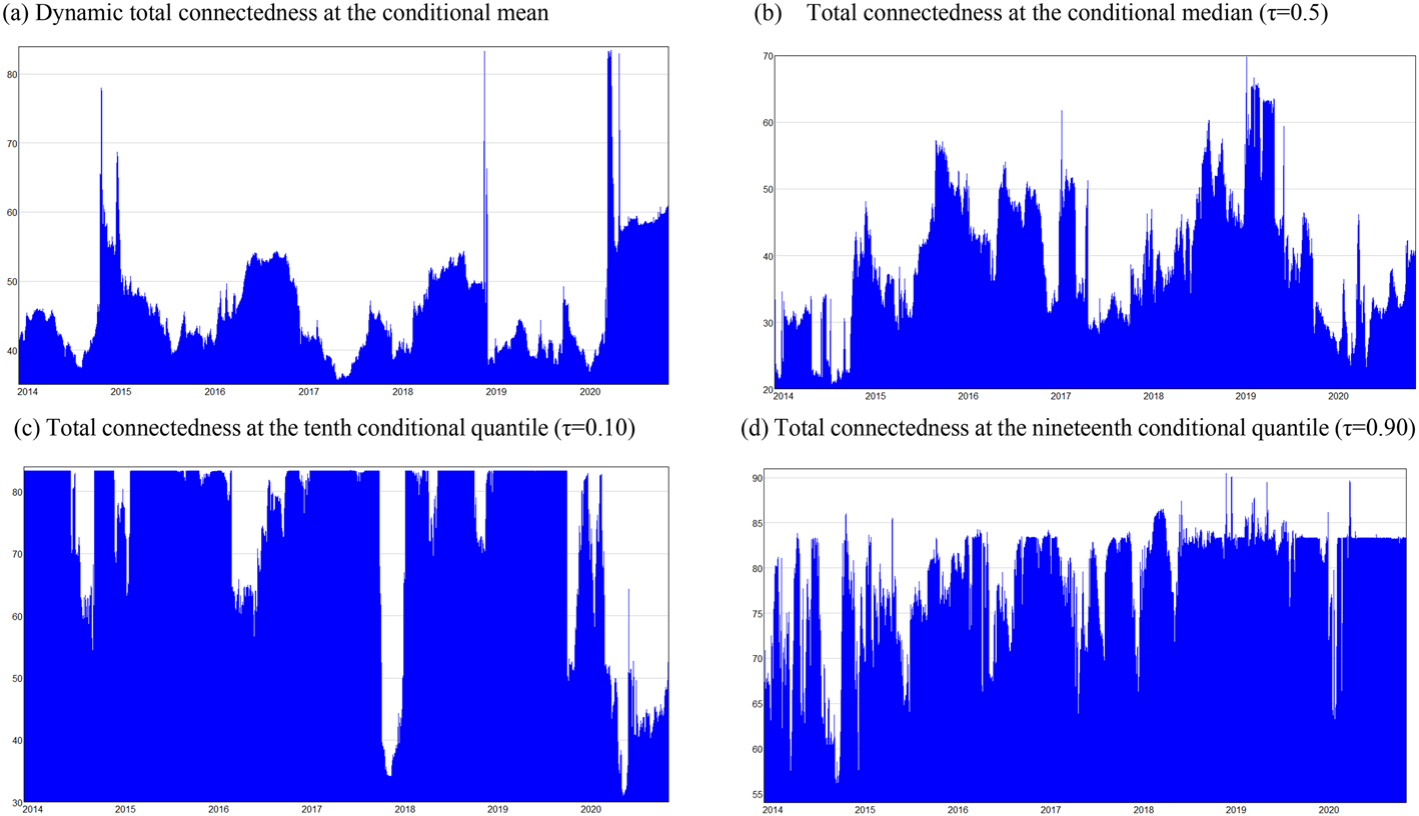
Dynamic total connectedness at the mean and selected quantiles of the distribution. **a** Dynamic total connectedness at the conditional mean. **b** Total connectedness at the conditional median (τ = 0.5). **c** Total connectedness at the tenth conditional quantile (τ = 0.10). **d** Total connectedness at the nineteenth conditional quantile (τ = 0.90)

To examine the dynamics of the system, we plot the time-varying evolution of the TSI evaluated at the average connectedness, namely at the conditional mean, median (τ = 0.50), at the left-tail dependence (τ = 0.10) and the right-tail dependence (τ = 0.90) of the conditional distribution (Fig. 3). We observe a large variation in total connectedness index, which is responsive to exogenous adverse shocks, namely the fiscal crisis (2015), the pre-election era elections (2018–2019) and the outbreak of the coronavirus pandemic (2020). Specifically, the spillover effects evaluated at the conditional mean and the median exhibit broadly similar behaviour, increasing as a result of the aforementioned exogenous shocks. Similarly, positive shocks such as the adoption of a new Economic Adjustment Programme after mid-2015, aimed at restoring the macroeconomic imbalances of the economy caused the mean and the median TSI to increase gradually. Periods without shocks or periods during which shocks are gradually fully absorbed, like the period after the elections of 2019, cause the TSI to gradually fall. The findings point to a rise in the sensitivity of the system to both extreme negative and positive shocks, as in Ando et al. (2018). As the energy variables are the driving forces in relation to hirings and firings, we conclude that the energy variables are more likely to affect the labour market during period of positive (right-tail-dependence) or negative (left-tail-dependence) shocks. Return to normal political and economic activity, namely periods without shocks or periods during which shocks are absorbed, results in a gradual fall of the TSI, indicating a lower magnitude of spillover transmission from energy variables to labour market outcomes. As in Kocaarslan et al. (2020) we find asymmetric interactions between oil prices, oil price uncertainty, interest rates and labour market outcomes. Further, the findings of the analysis suggest that shocks that emerge in international energy market are more likely to affect a small, oil-dependent economy during a recessionary and a flourishing economy. Consequently, the energy variables are more likely to transmit spillover effects during adverse and beneficial disturbances.

#### Net quantile dynamic spillover effects

**Fig. 4:**
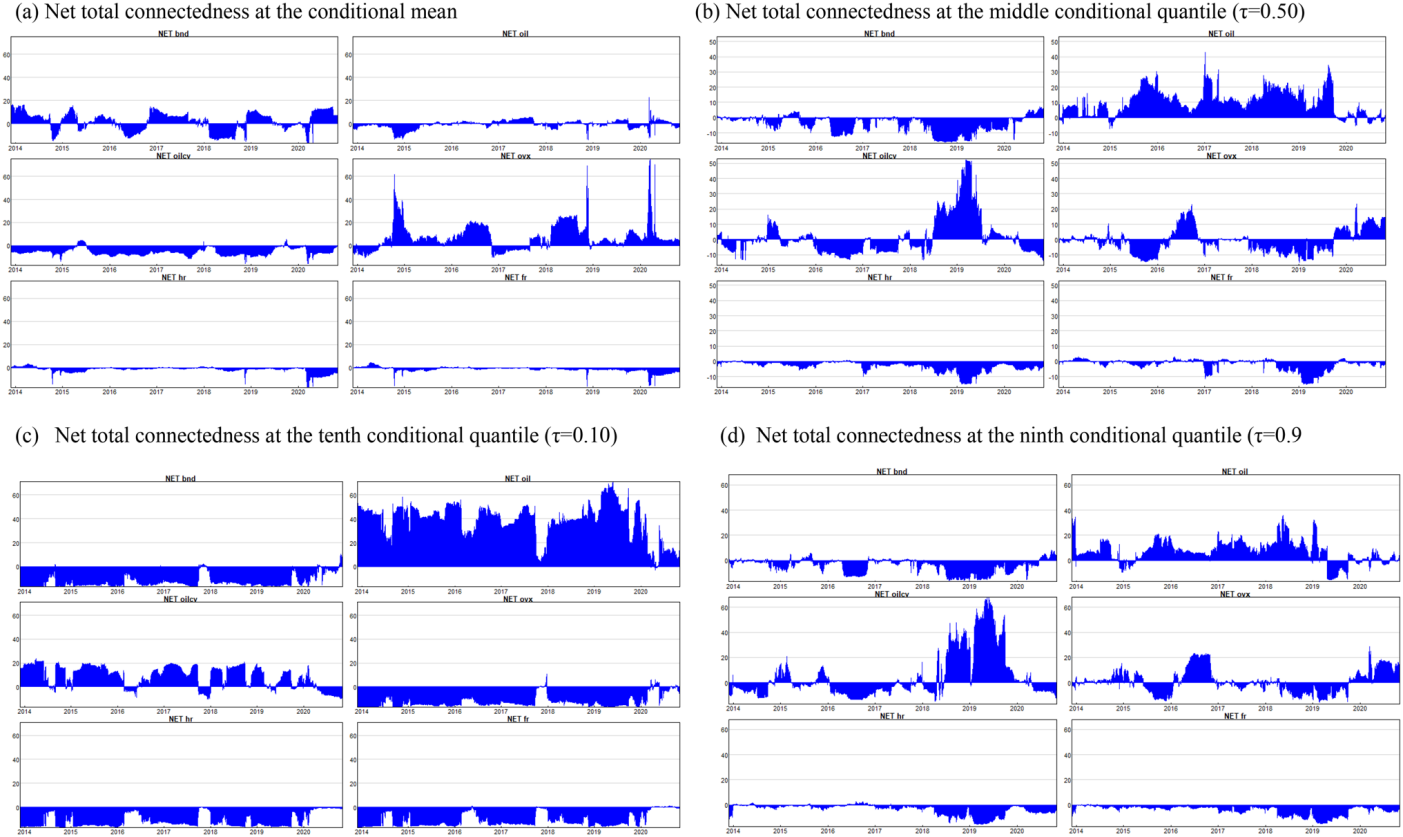
Net total connectedness at the mean and selected quantiles of the distribution. **a** Net total connectedness at the conditional mean. **b** Net total connectedness at the middle conditional quantile (τ = 0.50). **c** Net total connectedness at the tenth conditional quantile (τ = 0.10). **d** Net total connectedness at the ninth conditional quantile (τ = 0.9)

Similar patterns can be derived from the time-varying evolution of the net total directional connectedness at the median quantile (τ = 0.50), the left-tail dependence (τ = 0.10) and the right-tail dependence (τ = 0.90) (Fig. 4). Specifically, in all quantiles, including the conditional mean, the time-varying net total directional connectedness of hirings and firings is overall negative, suggesting the endogeneity of the variables of the system. This evidence is of great interest, as it adds further evidence on the endogeneity of the labour market variables and the exogeneity of the energy variables. Our findings suggest that the labour market reforms implemented have increased labour market flexibility and mobility. Further, our results indicate an upward trend in the net directional connectedness of firings and hirings after the COVID-19 outbreak, except for the left-tail-dependence quantile, where a decrease of the corresponding index is reported. These results indicate, besides a strong effect due to the COVID-19 crisis, the impact of the government measures being implemented in Greece to restrict the spread of the coronavirus disease. It is obvious that the effects of these restrictions are, as expected, more profound in the left-tail dependence of the conditional distribution, where we observe a diminishing impact of the variables of the system on firings and hirings after COVID-19 outbreak. These findings can be justified by the fact that the state intervention to sustain the pandemic was strenuous, and might have outgrown the influence of other factors (i.e. energy variables). This clarifies the fact that the impact of the energy variables on the domestic labour market has become less intense. Similar findings are reported in Betcherman et al. (2020) that show that the measures taken by the government to alleviate the effects of the COVID-19 lockdown have decreased separations. We observe that the net directional connectedness of the oil price variable is positive for the left and right tail of the conditional distribution and the middle quantile, for the period before the COVID-19 outbreak. However, after the outbreak it either decreases or even becomes net spillover effects receiver. A similar pattern, after the COVID-19 outbreak, can be identified when it comes to conditional volatility of oil prices. Generally, the net total directional connectedness of the 10-year bond rate and of the conditional volatility of oil prices evaluated at the mean are of different sign or present mixed spillover effects relative to the results of the quantile time-varying analysis. The latter finding, as in Ando et al. (2018) and Bouri et al. (2021) suggests that network models estimated at the conditional mean are unlikely to adequately capture the extent and the sign of variation of the spillover effects observed when large shocks occur and quantile estimation on a dynamic basis is of vital importance to provide in-depth analysis of the spillover transmission mechanisms.

### Robustness analysis

**Fig. 5:**
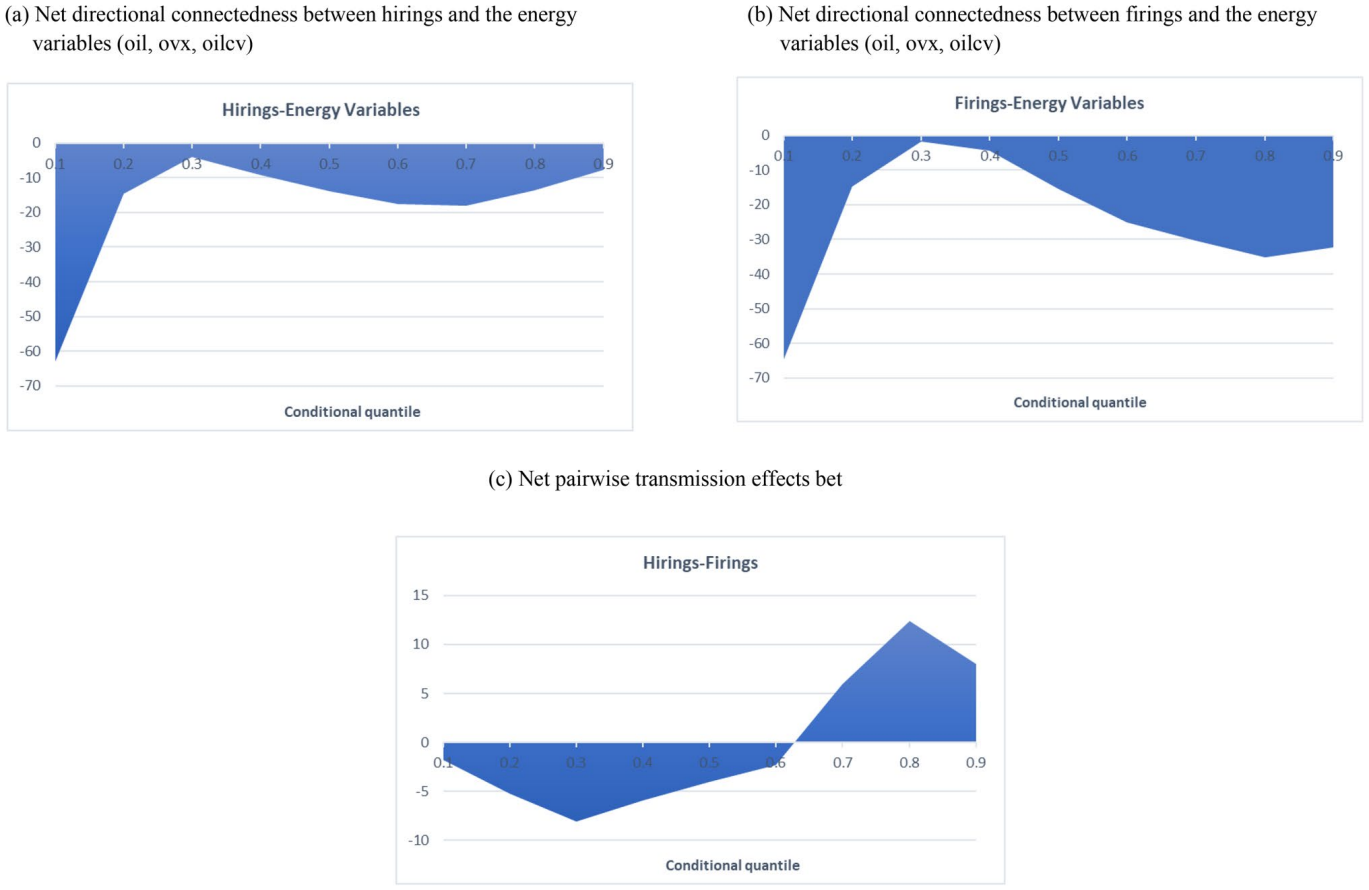
Net directional connectedness between hirings, firings and the energy variables (oil, ovx, oilcv) evaluated across different quantiles. **a** Net directional connectedness between hirings and the energy. **b** Net directional connectedness between firings and the energy variables (oil, ovx, oilcv) variables (oil, ovx, oilcv). **c** Net pairwise transmission effects between hirings and firings

Figure 5 depicts the net directional connectedness across the distribution between hirings and the energy variables (Fig. 5a), firings and the energy variables (Fig. 5b) and hirings and firings (Fig. 5c), respectively. Along the whole conditional distribution the evolution of the net directional connectedness between the labour market and energy variables, follows a similar pattern, indicating that the energy variables are the net transmitters of the spillover effects while hirings and firings are the endogenous variables. Further, for the left- and right-tail dependence of the conditional distribution the net spillover effects from the energy variables to hirings and firings, respectively, are higher at the left- and right-tail dependence, rather than at the middle quantiles. These findings indicate that positive and negative shocks in the energy market are more likely to affect the economy, during a recessionary (left-tail dependence) and a flourishing economy (right-tail dependence), rather than a normal economy (conditional median).

Concerning the pairwise transmission of spillover effects between hirings and firings there is evidence that up to the sixth quantile (τ = 0.6), firings is the net spillover transmitter while the corresponding net spillover impact from firings to hirings reaches its highest value (in absolute terms) at the τ = 0.3 quantile (8.1%) (Fig. 5c). After the sixth quantile (τ = 0.6), hirings become the net pairwise transmitter of the system, thus the driving force reaching its highest value at τ = 0.8 quantile (12.4%). This finding suggests that during recessionary (negative shocks) and normal economy periods firings is the driving force in its relationship with hirings, while the latter becomes the driving force during flourishing economy (positive shocks) periods.

Overall, the above findings add to the existing literature in that they shed light on the spillover effects of oil price changes on labour market outcomes, using a quantile connectedness analysis which is a unified methodology for measuring volatility spillover effects along the distribution. We find evidence that changes in energy markets asymmetrically affect the Greek labour market in recessionary and flourishing states of the economy, rather than normal times, a finding which is in line with Kocaarslan et al. (2020) for U.S., Raifu et al. (2020) for Nigeria and Cheratian et al. (2021) for the Middle East and North Africa region. Our analysis adds to the previous research as it examines the impact of oil price changes on the disaggregated components of labour market, namely on firings and hirings.

## Conclusion

Although the bulk of the empirical analysis has documented a negative relationship between oil prices and economic activity, there is a dearth of research on the presence of interrelations between oil prices, oil price volatility and employment and unemployment. In this paper we apply static and dynamic quantile connectedness methodology, using a quantile-based approach of Diebold and Yilmaz (2012) and Ando et al. (2018), to examine the spillover effects transmission mechanism between oil prices, oil price uncertainty, oil price volatility and interest rates on labour market in Greece. Greece is an interesting case to examine as it depends on oil imports and since 2010 has executed economic adjustment programmes to deal with the chronic deficiencies of the economy and has implemented significant reforms in the labour market. Moreover, following the COVID-19 outbreak the government has implemented restrictions to deal with the pandemic and taken measurers to alleviate the effects of the lockdown. The dynamic quantile connectedness estimation, adopted in the present analysis, provides a more comprehensive in-depth analysis than the ordinary mean approach, in the presence of conditional heterogeneity and departures from the Gaussian conditions, at the tails of the distribution, that is during a recessionary and/or a flourishing economy.

Overall, from the empirical analysis it is shown that both the mean and median spillover indices, which are measures of the average connectedness of the system, display broadly similar behaviour. There is evidence that the oil price variable is the most influential node of the energy variables on hirings and firings, followed by the conditional volatility of oil price changes and the crude oil volatility index. However, as conditional mean and median analysis may disguise spillover effects across the distribution, we apply quantile connectedness analysis with emphasis on the connectedness at the lower and upper tails of the distribution. The TSI exhibits a large variation across the quantiles, providing evidence in favour of excess spillover effects during negative (left-tail-dependence) and positive (right-tail-dependence) shocks. These findings suggest that changes in energy markets asymmetrically affect the Greek labour market in recessionary and flourishing states of the economy, rather than normal times.

Accordingly, we conduct a rolling analysis based on the quantile VAR to capture the volatility spillovers across the whole conditional distribution and examine the evolution of time-varying net total directional connectedness. The econometric analysis shows a large variation of the total spillover index, which is responsive to exogenous adverse and beneficial shocks. Overall, concerning the left and right-tail dependence of the conditional distribution, there is evidence of a different behaviour relative to the mean and the middle quantile, revealing a substantial higher time-varying connectedness of the system at the tails of the distribution.

Similar patterns can be derived from the time-varying evolution of the net total directional connectedness across the distribution at the median quantile (τ = 0.50), the left-tail dependence (τ = 0.10) and the right-tail dependence (τ = 0.90). In all quantiles, including the conditional mean, the time-varying net total directional connectedness suggests the endogeneity of the labour variables of the system and the exogeneity of the energy variables. Further, our results indicate an upward trend in the net directional connectedness of firings and hirings after the COVID-19 outbreak, except for the left-tail-dependence quantile, where a decrease of the corresponding index is reported. These results indicate a strong effect due to the COVID-19 pandemic and the state intervention to sustain the pandemic on the labour market that have outgrown the impact of the energy variables.

Most importantly our analysis shows that network of connectedness evaluated at the conditional mean is not suitable to reflect the degree of connectedness spillovers in the presence of beneficial and/or adverse shocks. Therefore, in a case of a system of variables which is characterized by significant shocks, quantile estimation on a dynamic basis is of vital importance to provide in-depth analysis of the spillover transmission mechanisms.

The results presented in our analysis have strong economic and social implications, especially given the recent global energy crisis. Rising energy prices could negatively affect production and competitiveness and ultimately disturb output growth. Further, they could result in negative labour market outcomes, with varying effects and intensity across sectors and vulnerable groups, such as the long-term unemployed, youth, females and especially poorer households. Given the severity of the energy shock, policy response is vital and should be based on an extensive and careful assessment of fiscal and welfare nexus and the country-specific macroeconomic stance.

The main limitation of the analysis is related to limited data availability. Subject to data availability in the future, the analysis can be extended to further research by utilizing additional control variables. However, the present framework could be applied to other medium-sized countries with a high degree of energy dependence on oil or to a panel of countries sharing the same characteristics.

## Data Availability

The data employed in this article stem from the ERGANI database published by the Ministry of Labour and Social Affairs for the labour market variables, the Brent crude oil spot prices from the Energy Information Administration (EIA), the crude oil price volatility index from options markets and the yield on 10-year Greek bonds from the Bank of Greece. They can be downloaded from the respective websites. We can provide the codes to replicate the results upon request.

## Footnotes

1 Following Kocaarslan et al. (2020) we utilize the implied price volatility and the conditional volatility of oil prices in the estimation procedure. However, we estimate models that the implied price volatility and the conditional volatility of oil prices enter separately in the model estimation. The results remain qualitatively the same and are available from the authors upon request.

2 We perform various robustness test using alternative window length and forecast horizon, showing qualitatively, similar patterns. Results are available upon request.
